# Dietary supplementation with Macleaya cordata extract alleviates intestinal injury in broiler chickens challenged with lipopolysaccharide by regulating gut microbiota and plasma metabolites

**DOI:** 10.3389/fimmu.2024.1414869

**Published:** 2024-07-19

**Authors:** Xiaohui Wang, Tong Zhang, Wenli Li, Ming’ai Zhang, Lianwen Zhao, Nianxue Wang, Xiaowen Zhang, Beibei Zhang

**Affiliations:** College of Animal Science and Technology, Qingdao Agricultural University, Qingdao, China

**Keywords:** Macleaya cordata extract, lipopolysaccharide, intestinal injury, gut microbiota, plasma metabolites, broiler chicken

## Abstract

**Introduction:**

The prevention and mitigation of intestinal immune challenge is crucial for poultry production. This study investigated the effects of dietary Macleaya cordata extract (MCE) supplementation on the prevention of intestinal injury in broiler chickens challenged with lipopolysaccharide (LPS).

**Methods:**

A total of 256 one-day-old male Arbor Acres broilers were randomly divided into 4 treatment groups using a 2×2 factorial design with 2 MCE supplemental levels (0 and 400 mg/kg) and 2 LPS challenge levels (0 and 1 mg/kg body weight). The experiment lasted for 21 d.

**Results and discussion:**

The results showed that MCE supplementation increased the average daily feed intake during days 0-14. MCE supplementation and LPS challenge have an interaction on the average daily gain during days 15-21. MCE supplementation significantly alleviated the decreased average daily gain of broiler chickens induced by LPS. MCE supplementation increased the total antioxidant capacity and the activity of catalase and reduced the level of malondialdehyde in jejunal mucosa. MCE addition elevated the villus height and the ratio of villus height to crypt depth of the ileum. MCE supplementation decreased the mRNA expression of pro-inflammatory cytokines interleukin (*IL*)*-6* and *IL-8* in the jejunum. MCE addition mitigated LPS-induced mRNA up-expression of pro-inflammatory factors *IL-1β* and *IL-17* in the jejunum. MCE supplementation increased the abundance of probiotic bacteria (such as *Lactobacillus* and *Blautia*) and reduced the abundance of pathogenic bacteria (such as *Actinobacteriota*, *Peptostretococcaceae*, and *Rhodococcus*), leading to alterations in gut microbiota composition. MCE addition altered several metabolic pathways such as Amino acid metabolism, Nucleotide metabolism, Energy metabolism, Carbohydrate metabolism, and Lipid metabolism in broilers. In these pathways, MCE supplementation increased the levels of L-aspartic acid, L-Glutamate, L-serine, etc., and reduced the levels of phosphatidylcholine, phosphatidylethanolamine, thromboxane B2, 13-(S)-HODPE, etc. In conclusion, dietary supplementation of 400 mg/kg MCE effectively improved the growth performance and intestinal function in LPS-challenged broiler chickens, probably due to the modulation of gut microbiota and plasma metabolites.

## Introduction

The intestine is a key site for nutrient absorption and immune response in broiler chickens. However, many factors such as pathogens, mycotoxins and heat stress can damage the intestines of broiler chickens. Lipopolysaccharide (LPS), a structural part of the outer membrane of Gram-negative bacteria, often results in intestinal immune challenge, thereby compromising intestinal integrity, nutrient transport and utilization (1, 2). LPS is recognized by the LPS binding protein, which launches an intercellular cascade response, eventually leading to the release of pro-inflammatory cytokines. This process induces intestinal injury in animals (1, 3) and immune stress in cells (4). LPS is often used to establish the model of intestinal injury in various animals, such as mice (5), chickens (6) and pigs (1). Previous studies have reported that the negative effects induced by LPS in broilers can be mitigated by nutritional interventions, such as probiotics (7), polysaccharides (8), and natural plant extracts (9).

Macleaya cordata, a medicinal plant belonging to the Papaveraceae family, is traditional Chinese medicine and has been widely used as a natural feed additive in animal husbandry in China, Europe, and North America (10, 11). Numerous studies have shown that Macleaya cordata extract (MCE) decreases inflammation, increases antioxidant capacity, and improves intestinal health, thereby increasing the growth performance of livestock and poultry (10, 12, 13). The main biologically active components of MCE are sanguinarine and chelerythrine, both of which belong to the quaternary benzo[c]phen-anthridine (14). Sanguinarine and chelerythrine are reported to have physiological effects, including anti-inflammatory, anti-oxidative, antimicrobial and antiviral properties (15, 16). It has been reported that MCE increases the body weight gain, average daily gain (ADG), and average daily feed intake (ADFI) and decreases the feed-to-gain ratio (F/G) in broiler chickens (10, 12, 17). In addition, MCE enhances the antioxidant function of mice challenged with enterotoxigenic Escherichia coli, as indicated by elevated activities of superoxide dismutase (SOD), catalase (CAT), and glutathione peroxidase (GSH-Px), and decreased levels of malondialdehyde (MDA) (18). Moreover, supplementation of MCE increases the villus height and the villus height to crypt height ratio (VCR) and decreases the crypt depth in the jejunum of broiler chickens with necrotic enteritis (19). MCE supplementation regulates serum immunological indices, such as decreasing the serum TNF-α and IL-6 levels of laying hens (20), and increasing the serum IL-10, TGF-β and IgG levels of neonatal piglets (21). However, it is unclear whether MCE can relieve the detrimental effects of LPS on the intestinal function of broiler chickens.

The gastrointestinal tract is not only the largest immunological organ but also the primary site of digestion and absorption for animals (22). The microbes in the gut can be influenced by pathogens, inflammatory cytokines, diet, exercise, gastrointestinal peptides, and so on (23). There is a dynamic balance in the gut microbiota, and when the balance is broken, it will lead to dysbiosis, which can influence the host immunity, the development of the gastrointestinal tract, and the health status of animals (24, 25). It has been reported that MCE changes the composition of gut microbiota, increases the abundance of *Lactobacillus*, and decreases the abundance of *Salmonella* and *Corynebacterium* (26, 27). At the same time, MCE can change the metabolic pathways of amino acids, vitamins and lipids, and increase the contents of butyrate and secondary cholic acid, thereby improving the growth performance and health status of broiler chickens (27). However, it remains unknown how MCE affects gut microbes and plasma metabolites in broilers challenged with LPS.

Therefore, the purpose of this study was conducted to investigate the effects of dietary MCE supplementation on growth performance, intestinal antioxidant function, intestinal morphology, and inflammation in broiler chickens challenged with LPS, and further to detect gut microbiota and plasma metabolites to reveal the underlying mechanism. Our study will be of significance for a more comprehensive understanding of the anti-immune stress mechanism of MCE in broiler chickens.

## Materials and methods

### Experimental design and animals

All experimental procedures were approved by the Animal Care and Use Committee of Qingdao Agricultural University. A total of 256 one-day-old male Arbor Acres broilers were obtained from a local commercial hatchery. The broilers were weighed and randomly assigned into 4 groups, including the CON group, MCE group, LPS group, and MCE+LPS group. Each group consisted of 8 replicates, with 8 broiler chickens per replicate. There was no significant difference in initial body weights among the groups. Chickens in the MCE and MCE+LPS groups were fed diets supplemented with MCE (400 mg/kg) from day 0 to day 21. Chickens in the LPS and MCE+LPS groups were intraperitoneally injected with LPS (1 mg/kg body weight) on days 15, 17, 19, and 21. Chickens in the CON and MCE groups were intraperitoneally injected with an equal volume of sterile saline. MCE was provided by Hunan Micolta Biological Resources Co., Ltd. The contents of two active ingredients, sanguinarine and chelerythrine, are 1.5% and 0.075%, respectively. LPS was purchased from Sigma (L-2880, *E. coli* serotype O55:B5). The broilers were reared in wire-floored cages under a 23-h photoperiod and had free access to feed and water. The room temperature was 34°C for the first week and then reduced by 2°C each week. The diet composition and nutritional levels are listed in **Table 1**.

**Table 1:** Composition and nutrient levels of basal diet (air-dry basis).

Ingredients	Contents, %	Nutrition level*^c^*	
Corn	55.69	ME, MJ/kg	12.58
Soybean meal, 46% CP	36.74	CP, %	21.53
Soybean oil	3.40	Ca, %	1.01
Limestone	1.15	Non-phytate phosphorus, %	0.46
Calcium hydrophosphate	2.10	Lys, %	1.23
NaCl	0.30	Met, %	0.52
D, L-Met, 98%	0.18	Thr, %	0.84
Vitamin premix^a^	0.10	Arg, %	1.47
Mineral premix*^b^*	0.15		
Choline chloride, 70%	0.18		
Antioxidant	0.01		
Total	100.00		

^a^Provided per kilogram of complete diet: vitamin A, 8,000 IU; vitamin D_3_, 2500 IU; vitamin E, 20 IU; vitamin K_3_, 2 mg; vitamin B_1_, 2 mg; vitamin B_2_, 6 mg; vitamin B_6_, 4.5mg; pantothenic acid, 12 mg; vitamin B_12_, 0.02 mg; niacin, 50 mg; folic acid, 1 mg and biotin, 0.15 mg.

^b^Provided per kilogram of complete diet: Mn, 100 mg; Fe, 80 mg; Zn, 75 mg; Cu, 8 mg; I, 0.4 mg; and Se, 0.3 mg

^c^Calculated value.

### Sample collection

Chickens (one bird per replicate) with average body weight were randomly selected and slaughtered at 3 h after LPS injection on day 21. Blood samples were collected using sterile blood collection tubes containing sodium heparin anticoagulant before slaughtering. Heparin plasma was collected after centrifugation at 3000 rpm for 10 min at 4°C, aliquoted, and stored at -20°C until analysis. After blood collection, the broilers were sacrificed by exsanguination. The ileum was collected and fixed in 4% paraformaldehyde for intestinal morphology analysis. The jejunum was collected, immediately frozen in liquid nitrogen, and stored at -80°C for gene expression analysis. Jejunal mucosa was scraped by glass slides, rapidly frozen in liquid nitrogen, and stored at -20°C for antioxidant assays.

### Growth performance

Body weight and feed consumption for each replicate cage were recorded on days 14 and 21. ADG, ADFI and F/G during days 0-14 and days 15-21 were calculated.

### Antioxidant capacity

The activities of CAT (A007-1-1), GSH-Px (A005-1-2) and SOD (A001-1-1) and the levels of MDA (A003-1-1) and T-AOC (A015-1-2) in intestinal mucosa were determined using commercial kits according to the manufacturer’s protocols (Nanjing jiancheng Bioengineering Institute, Nanjing, China). Mucosal homogenates (10%) were prepared by homogenizing intestinal mucosa in ice-cold sterile saline. The homogenates were centrifuged at 3000 rpm for 10 min at 4°C, and the supernatants were then collected for further assays. The protein contents in the supernatants were quantified using a Bicinchoninic acid (BCA) protein assay kit (Cwbio, Beijing, China). Antioxidant indices in intestinal mucosa were expressed as units per milligram of protein in the sample.

### Intestinal morphology

The intestinal tissues were dehydrated stepwise with ethanol, embedded in paraffin, and sectioned (5 μm). The tissue slices were stained with hematoxylin and eosin and sealed with gum. Images were captured using a light microscope (DM2000 LED, Leica, Germany) and analyzed using ZEISS ZEN 2011 (Blue version). Villus height was determined from the tip of the villus to the villus-crypt junction, and crypt depth was defined as the depth of the invagination between adjacent villi. and the VCR was calculated by dividing the villus height by the crypt depth. For each section, ten pairs of villi and crypts were observed, and the average was calculated as the final value.

### Real-time quantitative PCR

Total RNA was extracted from the jejunum using Trizol reagent (Invitrogen, Carlsbad, CA). The purity and concentration of total RNA were determined with a nanophotometer (Nano Photometer NP80, Implen, Germany). Reverse transcription into cDNA was performed using a PrimeScript RT reagent kit with gDNA Eraser (Takara, Osaka, Japan). Quantitative real-time PCR was performed using TB Green^®^ Premix Ex TaqTM (Takara, Osaka, Japan) on a CFX96 Real-Time PCR Detection System (Bio-rad, USA). The reaction mixture (25 μL) contained 12.5 μL of TB Green Premix Ex Taq (Tli RNaseH Plus) (2X), 0.5 μL of forward and reverse primers (10 μmol/L), 2 μL of cDNA, and 9.5 μL of sterile nuclease-free water. The PCR cycling conditions were as follows: 95°C for 30 s, followed by 40 cycles of 95°C for 5 s and 60°C for 30 s. The specificity of the PCR products was assessed using a melting curve. The primer sequences for the target and reference genes are listed in **Table 2**. The relative mRNA expression levels of target genes were calculated using the 2^–ΔΔCt^ method, and the housekeeping gene *GAPDH* was used as an internal control.

**Table 2:** Sequences of the primers used for quantitative real-time PCR.

Gene name^a^	Accession number	Primer sequence^b^ (5′ to 3′)	Product size, bp
*IL-1β*	XM_015297469.1	F: ACTGGGCATCAAGGGCTA	131
		R: GGTAGAAGATGAAGCGGGTC	
*IL-6*	XM_015281283.1	F: CGCCCAGAAATCCCTCCTC	152
		R: AGGCACTGAAACTCCTGGTC	
*IL-8*	XM_015301388.1	F: ATGAACGGCAAGCTTGGAGCTG	233
		R: TCCAAGCACACCTCTCTTCCATCC	
*IL-10*	NM_001004414.2	F: CGCTGTCACCGCTTCTTCA	88
		R: TCCCGTTCTCATCCATCTTCTC	
*IL-17*	NM_204460.1	F: CTCCGATCCCTTATTCTCCTC	292
		R: AAGCGGTTGTGGTCCTCAT	
		R: GCCAAGGTGTAGGTGCGATTCC	
*TNF-α*	XM_046927265.1	F: GAGCGTTGACTTGGCTGTC	64
		R: AAGCAACAACCAGCTATGCAC	
*IFN-γ*	NM_205149.2	F: AGCTGACGGTGGACCTATTATT	259
		R: GGCTTTGCGCTGGATTC	
*iNOS*	NM_204961.2	F: TGGGTGGAAGCCGAAATA	241
		R: GTACCAGCCGTTGAAAGGAC	
*GAPDH*	NM_204305.1	F: TGCTGCCCAGAACATCATCC	142
		R: ACGGCAGGTCAGGTCAACAA	

^a^IL, interleukin; TNF-α, tumor necrosis factor alpha, IFN-γ, interferon γ, iNOS, inducible nitric oxide synthase, and GAPDH, glyceraldehyde-3-phosphate dehydrogenase.

^b^F, forward; R, reverse.

### Gut microbiome

#### DNA extraction and high-throughput sequencing

Total microbial genomic DNA was extracted from ileal digesta using the E.Z.N.A.^®^ soil DNA Kit (Omega Bio-tek, Norcross, GA, U.S.) according to the manufacturer’s instructions. The quality and concentration of DNA were determined by 1.0% agarose gel electrophoresis and a NanoDrop^®^ ND-2000 spectrophotometer (Thermo Fisher Scientific, MA, USA). The hypervariable region V3-V4 of the bacterial 16S rRNA gene was amplified with primer pairs 338F (5’-ACTCCTACGGGAGGCAGCAG-3’) and 806R (5’-GGACTACHVGGGTWTCTAAT-3’) on an ABI GeneAmp^®^ 9700 PCR thermocycler (ABI, CA, USA). PCR amplification was performed using TransStart^®^ FastPfu DNA Polymerase kits (TransGen AP221-02) under the cycling conditions: 95°C for 3 min, followed by 27 cycles of 95°C for 30 s, 55°C for 30 s and 72°C for 45 s, and a final extension at 72°C for 10 min. All samples were amplified in triplicate. The PCR product was extracted from 2% agarose gel, purified using an AxyPrep DNA Gel Extraction kit (Axygen Biosciences, Union City, CA, USA) according to the manufacturer’s instructions, and quantified using the Quantus™ Fluorometer (Promega, USA). Purified amplicons were pooled in equimolar amounts and paired-end sequenced on an Illumina MiSeq PE300 platform (Illumina, San Diego, USA).

#### Sequence Processing and Bioinformatics Analysis

Raw FASTQ files were de-multiplexed using an in-house perl script, quality-filtered using fastp version 0.19.6, and merged using FLASH version 1.2.7. The optimized sequences were clustered into operational taxonomic units (OTUs) using UPARSE 7.1 at a 97% sequence similarity level. The taxonomy of each OTU representative sequence was analyzed using RDP Classifier version 2.2 against the 16S rRNA gene database (Silva v138) at a confidence threshold of 0.7. The α-diversity was analyzed using Mothur (1.30.2). The β-diversity and community barplot were analyzed using Qiime (1.9.1). The microbiota with linear discriminant analysis (LDA) scores > 2 were identified using the linear discriminant analysis effect size (LEfSe) method.

#### Detection of plasma metabolite composition by non-targeted metabolomics

To extract metabolites, 100 μL of plasma sample was mixed with 400 μL of extraction solution (acetonitrile: methanol = 1: 1, v/v) containing 0.02 mg/mL L-2-chlorophenylalanine as an internal standard, followed by vortexing for 30 s and sonication (40 kHz) for 30 min at 4°C. The mixture was incubated at -20°C for 30 min to precipitate the proteins. The mixture was centrifuged for 15 min (4°C, 13000 g). The supernatant was collected and blown dry under nitrogen. The samples were then re-dissolved in 100 µL of aqueous acetonitrile (acetonitrile: water = 1: 1, v/v) and sonicated (40 kHz)) for 5 min at 5°C, followed by centrifugation (13000 g, 4°C) for 10 min. The supernatant was collected and subjected to LC-MS/MS analysis. Quality control (QC) samples were analyzed at regular intervals (every 12 samples).

The LC-MS/MS analysis was conducted on a Thermo UHPLC-Q Exactive HF-X system equipped with an ACQUITY HSS T3 column (100 mm × 2.1 mm, i.d., 1.8 μm; Waters, USA) at Majorbio Bio-Pharm Technology Co. Ltd. (Shanghai, China). The mobile phases consisted of 0.1% formic acid in Solvent A (water: acetonitrile = 95: 5, v/v) and 0.1% formic acid in Solvent B (acetonitrile: isopropanol: water = 47.5: 47.5: 5, v/v/v). The flow rate was 0.40 mL/min, and the column temperature was 40°C. The injection volume was 3 μL. The LC-MS/MS data were acquired using a Thermo UHPLC-Q Exactive HF-X Mass Spectrometer equipped with an electrospray ionization (ESI) source operated in both positive and negative ion modes. The optimal conditions were set as follows: source temperature at 425°C; sheath gas flow rate at 50 arb; Aux gas flow rate at 13 arb; ion-spray voltage floating (ISVF) at -3 500 V in the negative ion mode and 3 500 V in the positive ion mode, respectively; Normalized collision energy, 20-40-60 V rolling for MS/MS. Full MS resolution was 60000, and MS/MS resolution was 7500. Data acquisition was performed in the Data-Dependent Acquisition mode. The detection was carried out over a mass range of 70-1 050 m/z.

LC/MS raw data were preprocessed using Progenesis QI (Waters Corporation, Milford, USA) and analyzed on the Majorbio cloud platform (Majorbio Biotech, Shanghai, China). The principal component analysis (PCA) and partial least squares-discriminant analysis (PLS-DA) were performed using the R package “ropls” (version 1.6.2), and 7-cycle interactive validation was used to evaluate the stability of the model. Differential metabolites in volcano plots were identified based on *P* values (*P* < 0.05, t-test) and VIP values (VIP > 1). Differential metabolites among the two groups were mapped into their biochemical pathways through metabolic enrichment and pathway analysis based on the KEGG database (http://www.genome.jp/kegg/). Python package “scipy.stats” (https://docs.scipy.org/doc/scipy/) was used to perform enrichment analysis.

### Statistical analysis

Data are analyzed using SPSS version 26.0 (IBM Corp., Armonk, NY, USA) and expressed as mean ± SEM. Differences between groups were analyzed using a two-way analysis of variance (ANOVA), followed by Duncan’s multiple comparison test. The graphs were created using GraphPad Prism 8 Software (GraphPad Software Inc., La Jolla, CA, USA). Differences were considered statistically significant when *P* < 0.05.

## Results

### Growth performance

As shown in **Table 3**, MCE supplementation significantly increased the ADFI (*P* < 0.05) in broiler chickens, but did not affect the ADG and F/G (*P* > 0.05) during days 0-14. During days 15-21, there was an interaction between the LPS challenge and MCE supplementation on the ADG (*P* < 0.05). The LPS group significantly decreased the ADG in broiler chickens compared with the CON group, while the MCE+LPS group significantly suppressed the LPS-induced decrease in the ADG (*P* < 0.01). LPS challenge significantly decreased the ADFI in broiler chickens (*P* < 0.001) regardless of MCE supplementation. In addition, MCE supplementation, LPS challenge and their interaction had no effects on the F/G (*P* > 0.05) during days 0-21.

**Table 3:** Effects of Macleaya cordata extract (MCE) on growth performance in broiler chickens challenged with LPS.

Items		0-14d			15-21d		
		ADG, g	ADFI, g	F/G	ADG, g	ADFI, g	F/G
CON		21.58	29.88	1.39	48.06^a^	68.02	1.43
MCE		22.31	31.37	1.41	46.32^a^	69.37	1.50
LPS		21.04	29.50	1.40	39.72^c^	58.84	1.48
MCE+LPS		21.58	30.92	1.44	42.42^b^	61.40	1.45
SEM		0.274	0.356	0.014	0.704	1.158	0.017
Main effect							
MCE	–	21.31	29.69	1.39	43.89	63.43	1.46
	+	21.94	31.15	1.42	44.37	65.39	1.47
LPS	–	21.95	30.63	1.40	47.19	68.70	1.47
	+	21.31	30.21	1.42	41.07	60.12	1.47
*P* value							
MCE		0.261	0.044	0.300	0.534	0.266	0.668
LPS		0.258	0.549	0.487	<0.001	<0.001	0.973
Interaction		0.862	0.964	0.862	0.008	0.727	0.140

^a-c^Mean values in the same column with different lowercase letters indicate significant difference (P < 0.05).

CON group, broilers received a basal diet without a LPS challenge; MCE group, broilers received a basal diet supplemented with 400 mg/kg MCE without a LPS challenge; LPS group, broilers received a basal diet and were subjected to a LPS challenge; MCE+LPS group, broilers received a basal diet supplemented with 400 mg/kg MCE and were subjected to a LPS challenge; ADG, average daily gain; ADFI, average daily feed intake; F/G: feed to gain ratio. N = 8 in each group.

### Jejunal mucosal antioxidant status

As shown in **Table 4**, LPS challenge significantly decreased the T-AOC (*P* < 0.001) and the activities of CAT (*P* < 0.01) and SOD (*P* < 0.01) and increased the level of MDA (*P* < 0.05) in broiler chickens. MCE supplementation significantly increased the T-AOC and the activity of CAT and reduced the level of MDA in broiler chickens (*P* < 0.05). MCE supplementation, LPS challenge and their interaction have no effects on GSH-Px activity (*P* > 0.05).

**Table 4:** Effects of Macleaya cordata extract (MCE) on the antioxidant ability of jejunum in broiler chickens challenged with LPS.

Items		T-AOC, U/mgprot	CAT, U/mgprot	GSH-Px, U/mgprot	SOD, U/mgprot	MDA, nmol/mgprot
CON		0.15	2.05	10.07	484.78	0.33
MCE		0.17	2.60	10.86	524.99	0.25
LPS		0.09	1.56	12.00	363.23	0.42
MCE+LPS		0.12	1.76	13.12	431.83	0.30
SEM		0.008	0.110	0.741	18.117	0.021
Main effect						
MCE	–	0.12	1.81	11.04	424.01	0.37
	+	0.14	2.18	11.99	478.41	0.28
LPS	–	0.16	2.33	10.47	504.89	0.29
	+	0.11	1.66	12.56	397.53	0.36
*P*-value						
MCE		0.039	0.036	0.531	0.064	0.012
LPS		<0.001	0.001	0.176	0.001	0.046
Interaction		0.918	0.300	0.913	0.616	0.616

CON group, broilers received a basal diet without a LPS challenge; MCE group, broilers received a basal diet supplemented with 400 mg/kg MCE without a LPS challenge; LPS group, broilers received a basal diet and were subjected to a LPS challenge; MCE+LPS group, broilers received a basal diet supplemented with 400 mg/kg MCE and were subjected to a LPS challenge; T-AOC, total antioxidant capacity; CAT, catalase; GSH-Px, glutathione peroxidase; SOD, superoxide dismutase; MDA, malondialdehyde. N = 8 in each group.

### Ileal morphology

**Figure 1:**
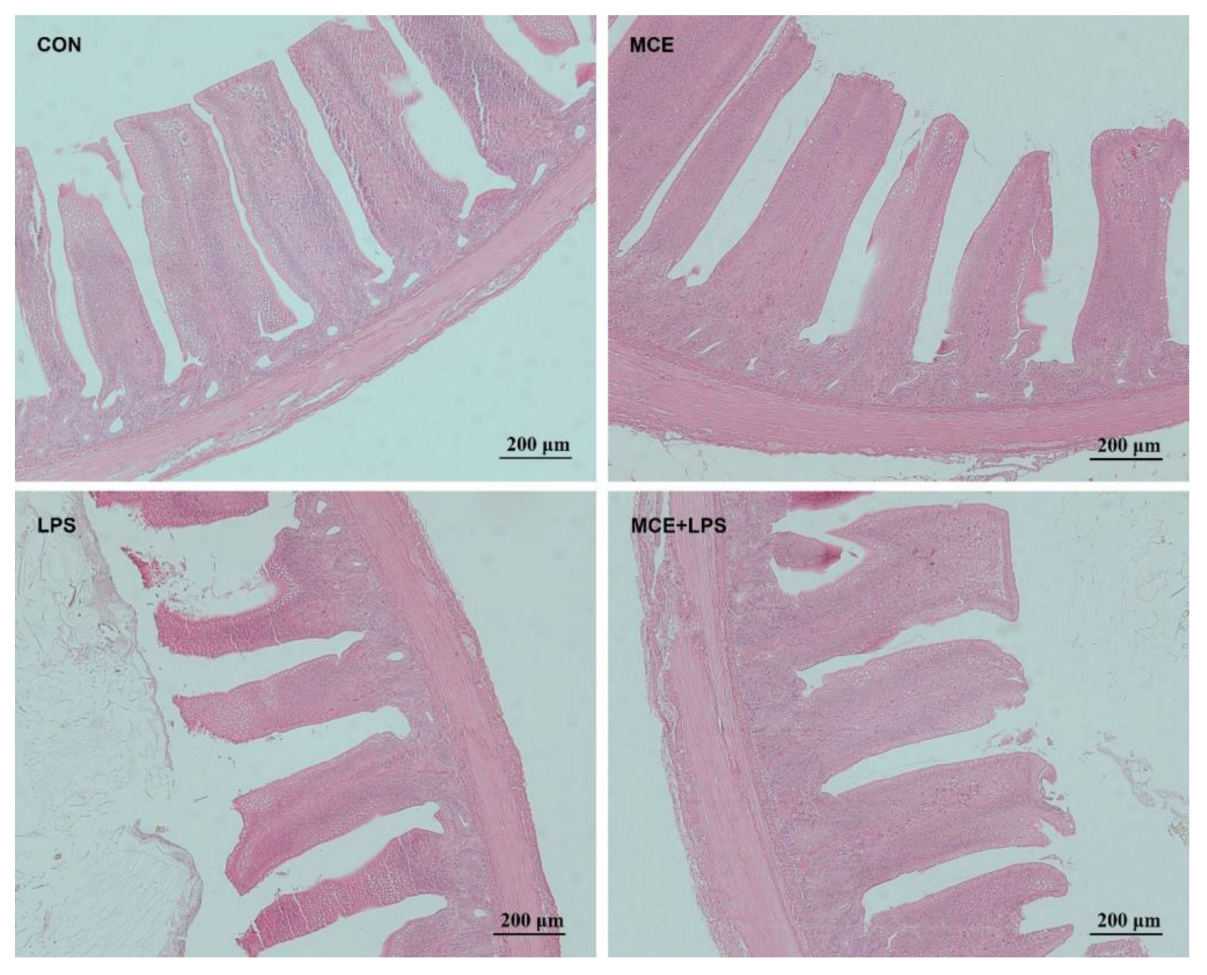
The representative images of ileal morphology by hematoxylin and eosin staining. Original magnification 100×, scale bar 200 μm. CON group, broilers received a basal diet without a LPS challenge; MCE group, broilers received a basal diet supplemented with 400 mg/kg MCE without a LPS challenge; LPS group, broilers received a basal diet and were subjected to a LPS challenge; MCE+LPS group, broilers received a basal diet supplemented with 400 mg/kg MCE and were subjected to a LPS challenge.

Hematoxylin and eosin staining results revealed nearly normal intestinal morphology in the CON and MCE groups. However, the LPS group exhibited significant intestinal mucosa damage, including damaged villus tips and villi atrophy. Nevertheless, intestinal mucosa damage was reduced in the MCE+LPS group (**Figure 1**). As shown in **Table 5**, LPS challenge significantly decreased the villus height in broiler chickens (*P* < 0.01), while MCE supplementation significantly increased the villus height (*P* < 0.05). LPS challenge significantly decreased the VCR in broiler chickens (*P* < 0.01), while MCE supplementation significantly increased the VCR (*P* < 0.05). MCE supplementation and LPS challenge have no effects on crypt depth (*P* > 0.05).

**Table 5:** Effects of Macleaya cordata extract (MCE) on the ileal morphology in broiler chickens challenged with LPS.

Items		Villus height, μm	Crypt depth, μm	VCR
CON		700.09	146.98	4.83
MCE		731.88	151.49	5.10
LPS		651.27	168.00	4.06
MCE+LPS		681.00	148.14	4.64
SEM		8.816	4.410	0.117
Main effect				
MCE	–	675.68	157.49	4.45
	+	706.44	149.82	4.87
LPS	–	715.98	149.23	4.96
	+	666.13	158.07	4.35
*P*-value				
MCE		0.046	0.389	0.046
LPS		0.002	0.322	0.005
Interaction		0.945	0.176	0.453

CON group, broilers received a basal diet without a LPS challenge; MCE group, broilers received a basal diet supplemented with 400 mg/kg MCE without a LPS challenge; LPS group, broilers received a basal diet and were subjected to a LPS challenge; MCE+LPS group, broilers received a basal diet supplemented with 400 mg/kg MCE and were subjected to a LPS challenge; VCR, villus height to crypt depth ratio. N = 8 in each group.

### Relative mRNA expression of jejunal inflammatory genes

As shown in **Table 6**, MCE supplementation and LPS challenge have interactions on the mRNA expression of *IL-1β* and *IL-17*. Compared with the CON group, the LPS group significantly increased the mRNA expression of *IL-1β* and *IL-17*, while the MCE+LPS group significantly suppressed the increase of mRNA expression of *IL-1β* and *IL-17* induced by LPS challenge (*P* < 0.01). LPS challenge significantly increased the mRNA expression of *IL-8* (*P* < 0.01), *IFN-γ* (*P* < 0.05), and *iNOS* (*P* < 0.05). MCE supplementation significantly decreased the mRNA expression of *IL-6* and *IL-*8 (*P* < 0.05).

**Table 6:** Effects of Macleaya cordata extract (MCE) on the jejunal relative mRNA expression of inflammatory genes in broiler chickens challenged with LPS.

Items		*IL-1β*	*IL-6*	*IL-8*	*IL-10*	*IL-17*	*TNF-α*	*IFN-γ*	*iNOS*
CON		1.01^c^	1.08	1.04	1.01	1.01^c^	1.01	1.06	1.03
MCE		1.14^c^	0.77	0.94	1.01	1.15^c^	0.89	0.98	1.03
LPS		2.50^a^	1.10	1.69	0.99	3.11^a^	1.04	1.39	1.27
MCE+LPS		1.74^b^	0.78	1.21	0.93	2.23^b^	1.01	1.59	1.12
SEM		0.141	0.064	0.080	0.035	0.177	0.031	0.097	0.039
Main effect									
MCE	–	1.76	1.09	1.36	1.00	2.06	1.03	1.22	1.15
	+	1.44	0.77	1.08	0.97	1.69	0.95	1.29	1.08
LPS	–	1.07	0.93	0.99	1.01	1.08	0.95	1.02	1.03
	+	2.12	0.94	1.45	0.96	2.67	1.02	1.49	1.19
*P*-value									
MCE		0.042	0.012	0.029	0.699	0.008	0.220	0.732	0.337
LPS		<0.001	0.896	0.001	0.503	<0.001	0.252	0.016	0.035
Interaction		0.007	0.965	0.134	0.716	0.001	0.512	0.436	0.279

^a-c^Mean values in the same column with different lowercase letters indicate significant difference (P < 0.05).

CON group, broilers received a basal diet without a LPS challenge; MCE group, broilers received a basal diet supplemented with 400 mg/kg MCE without a LPS challenge; LPS group, broilers received a basal diet and were subjected to a LPS challenge; MCE+LPS group, broilers received a basal diet supplemented with 400 mg/kg MCE and were subjected to a LPS challenge; IL, interleukin; TNF-α, tumor necrosis factor alpha; IFN-γ, interferon γ; iNOS, inducible nitric oxide synthase. N = 8 in each group.

### Gut microbiome

#### α-diversity and β-diversity

**Figure 2:**
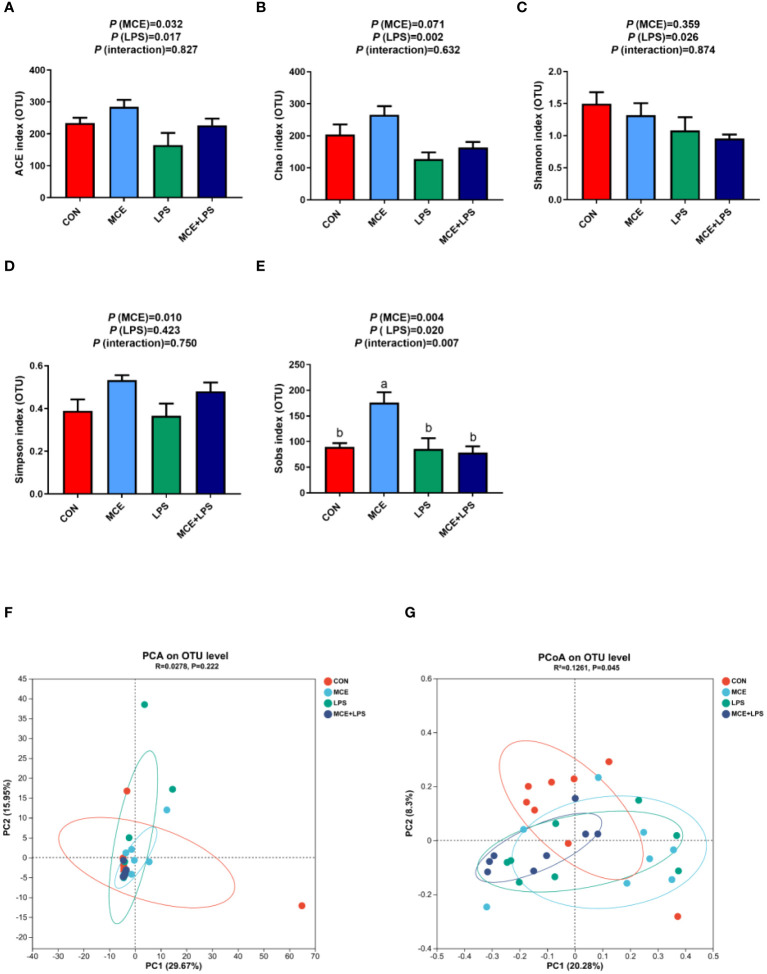
Effects of Macleaya cordata extract (MCE) on α-diversity and β-diversity of gut microbiota in broiler chickens challenged with LPS. Changes in α-diversity: **(A)** ACE index, **(B)** Chao index, **(C)** Shannon index, **(D)** Simpson index, and **(E)** Sobs index α-diversity. **(F)** PCA and **(G)** PCoA of the β-diversity. CON group, broilers received a basal diet without a LPS challenge; MCE group, broilers received a basal diet supplemented with 400 mg/kg MCE without a LPS challenge; LPS group, broilers received a basal diet and were subjected to a LPS challenge; MCE+LPS group, broilers received a basal diet supplemented with 400 mg/kg MCE and were subjected to a LPS challenge. ^a, b^ Mean values with no common lowercase letters indicate significant difference (*P* < 0.05). N = 8 in each group.

As shown in **Figures 2A–E**, LPS challenge significantly decreased the abundance-based coverage estimator (ACE) index (*P* < 0.05), Chao index (*P* < 0.01) and Shannon index (*P* < 0.05), while MCE supplementation significantly increased the ACE index (*P* < 0.05). MCE supplementation significantly elevated the Simpson index (*P* < 0.05) regardless of LPS challenge. There is an interaction on the Sobs index between MCE supplementation and LPS challenge (*P* < 0.05). The Sobs index was significantly higher in the MCE group than in other groups (*P* < 0.01). PCA and PCoA were used to examine the similarities and differences of gut microbiota between groups in broiler chickens and there was no distinct separation between groups (**Figures 2F, G**).

### The composition of gut microbiota and LEfSe analysis

**Figure 3:**
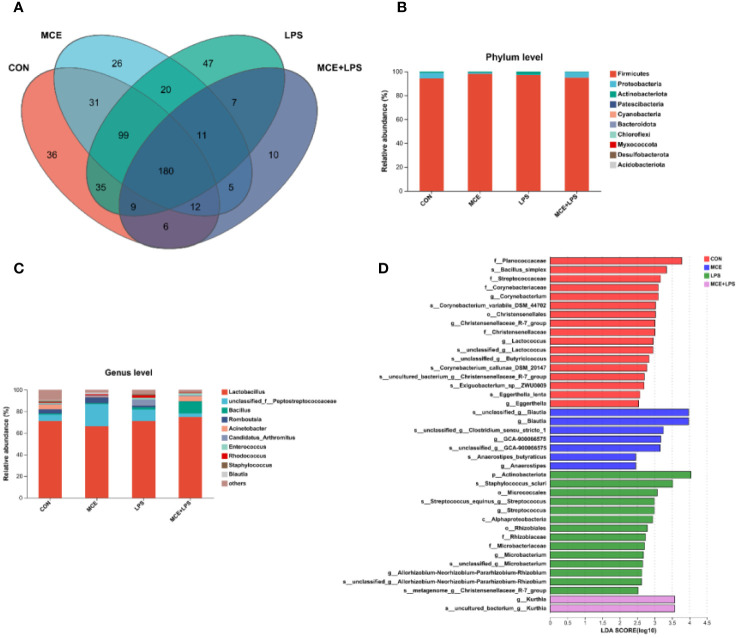
Effects of Macleaya cordata extract (MCE) on the composition of gut microbiota in broiler chickens challenged with LPS. **(A)** the Venn diagram of gut microbiota, **(B)** the composition of gut microbiota at the phylum level, **(C)** the composition of gut microbiota at the genus level, and **(D)** the LEfSe analysis of gut microbiota. CON group, broilers received a basal diet without a LPS challenge; MCE group, broilers received a basal diet supplemented with 400 mg/kg MCE without a LPS challenge; LPS group, broilers received a basal diet and were subjected to a LPS challenge; MCE+LPS group, broilers received a basal diet supplemented with 400 mg/kg MCE and were subjected to a LPS challenge. N = 8 in each group.

As illustrated in the Venn diagram, a total of 180 OTUs were identified in all groups, with 36, 26, 47 and 10 particular OTUs in the CON, MCE, LPS, and MCE+LPS groups, respectively (**Figure 3A**). The dominant microbiota in all groups mainly included *Firmicutes*, *Proteobacteria*, *Actinobacteriota*, *Patescibacteri*a at the phylum level (**Figure 3B**), and *Lactobacillus*, *Peptostreptococcaceae*, *Bacillus* and *Romboutsia* at the genus level (**Figure 3C**). The LDA scores were used to identify taxonomic biomarkers contributing to the differences between groups (**Figure 3D**). *Blautia*, *GCA-900066575*, *Anaerostipes butyraticus*, *Anaerostipes*, and *Clostridium_sensu_stricto_1* had higher LDA scores in the MCE group. *Actinobacteriota*, *Staphylococcus_sciuri*, *Micrococcales*, *Streptococcus*, *Alphaproteobacteria*, *Rhizobiales*, *Microbacterium*, *Rhizobiaceae*, *Christensenellaceae_R-7_group*, and *Allorhizobium-Neorhizobium-Pararhizobium-Rhizobium* had higher LDA scores in the LPS group. *Kurthia* had a higher LDA score in the MCE+LPS group.

### The relative abundance of gut microbiota at the phylum and genus levels

**Figure 4:**
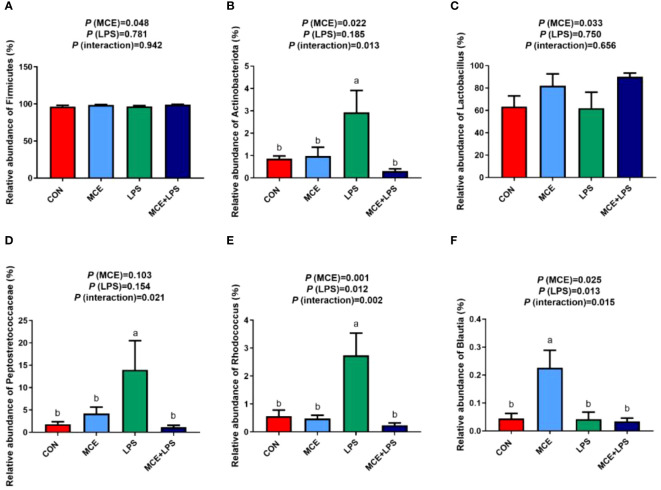
Effects of Macleaya cordata extract (MCE) on the relative abundance of bacteria at the phylum and genus levels in broiler chickens challenged with LPS. **(A)** *Firmicutes* and **(B)** *Actinobacteriota* at the phylum level. **(C)** *Lactobacillus*, **(D)** *Peptostretococcaceae*, **(E)** *Rhodococcus*, and **(F)** *Blautia* at the genus level. CON group, broilers received a basal diet without a LPS challenge; MCE group, broilers received a basal diet supplemented with 400 mg/kg MCE without a LPS challenge; LPS group, broilers received a basal diet and were subjected to a LPS challenge; MCE+LPS group, broilers received a basal diet supplemented with 400 mg/kg MCE and were subjected to a LPS challenge. ^a, b^ Mean values with no common lowercase letters indicate significant difference (*P* < 0.05). N = 8 in each group.

As depicted in **Figures 4A, B**, at the phylum level, MCE supplementation significantly increased the relative abundance of *Firmicutes* (*P* < 0.05). Compared with the CON group, the LPS group significantly increased the relative abundance of *Actinobacteriota*, while the MCE+LPS group significantly suppressed the increase induced by LPS challenge (*P* < 0.05). As shown in **Figures 4C–F**, at the genus level, MCE supplementation significantly increased the relative abundance of *Lactobacillus* (*P* < 0.05). Interactions have been observed in the relative abundance of *Peptostretococcaceae*, *Rhodococcus* and *Blautia* between MCE supplementation and LPS challenge (*P* < 0.05). Compared with the CON group, the LPS group significantly increased the relative abundance of *Peptostretococcaceae* and *Rhodococcus*, while the MCE+LPS group suppressed the increase induced by LPS challenge (*P* < 0.05). *Blautia* had a higher relative abundance in the MCE group than in other groups (*P* < 0.05).

### Non-targeted metabolomics in plasma

#### PCA and PLS-DA plots

**Figure 5:**
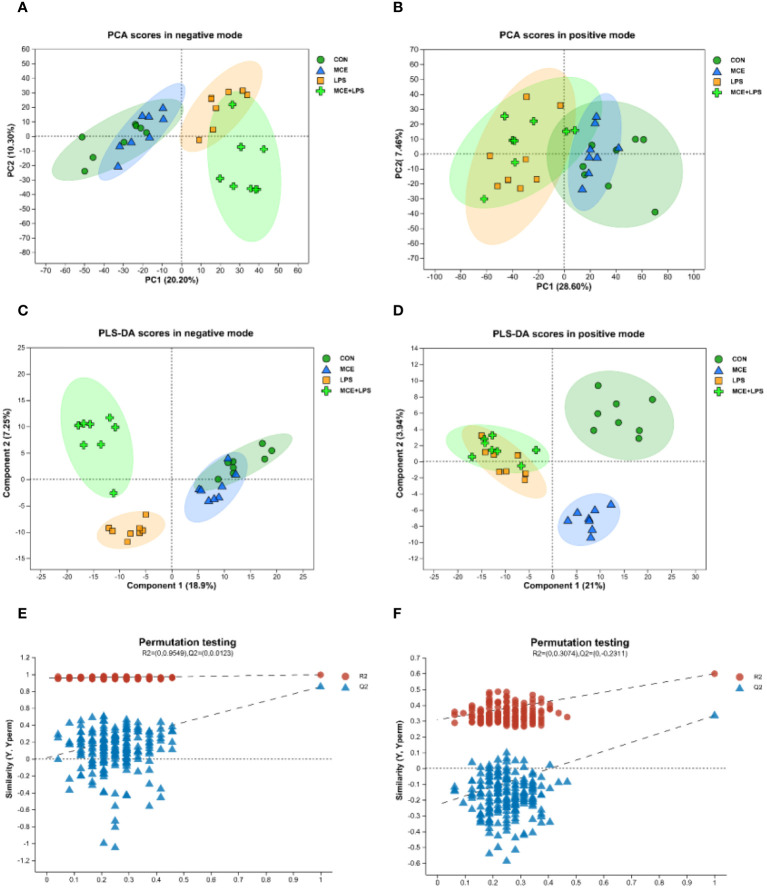
PCA and PLS-DA score plots of plasma metabolites in broiler chickens. **(A, B)** PCA scores in negative and positive modes. **(C, D)** PLS-DA scores in negative and positive modes. **(E, F)** Permutation test plots of PLS-DA scores in negative and positive modes. CON group, broilers received a basal diet without a LPS challenge; MCE group, broilers received a basal diet supplemented with 400 mg/kg MCE without a LPS challenge; LPS group, broilers received a basal diet and were subjected to a LPS challenge; MCE+LPS group, broilers received a basal diet supplemented with 400 mg/kg MCE and were subjected to a LPS challenge. N = 8 in each group.

There were 551 metabolites identified in the positive mode and 217 metabolites identified in the negative mode in all groups. The differences in metabolites between groups are shown in the PCA and PLS-DA plots (**Figure 5**). There was a distinct separation between the CON group and the LPS group in both positive and negative modes. A clear separation was also observed in the negative mode between the LPS and MCE+LPS groups. There was a clear separation between the CON and MCE groups in PLS-DA score plots in the positive mode.

### Alteration in plasma metabolic composition

**Figure 6:**
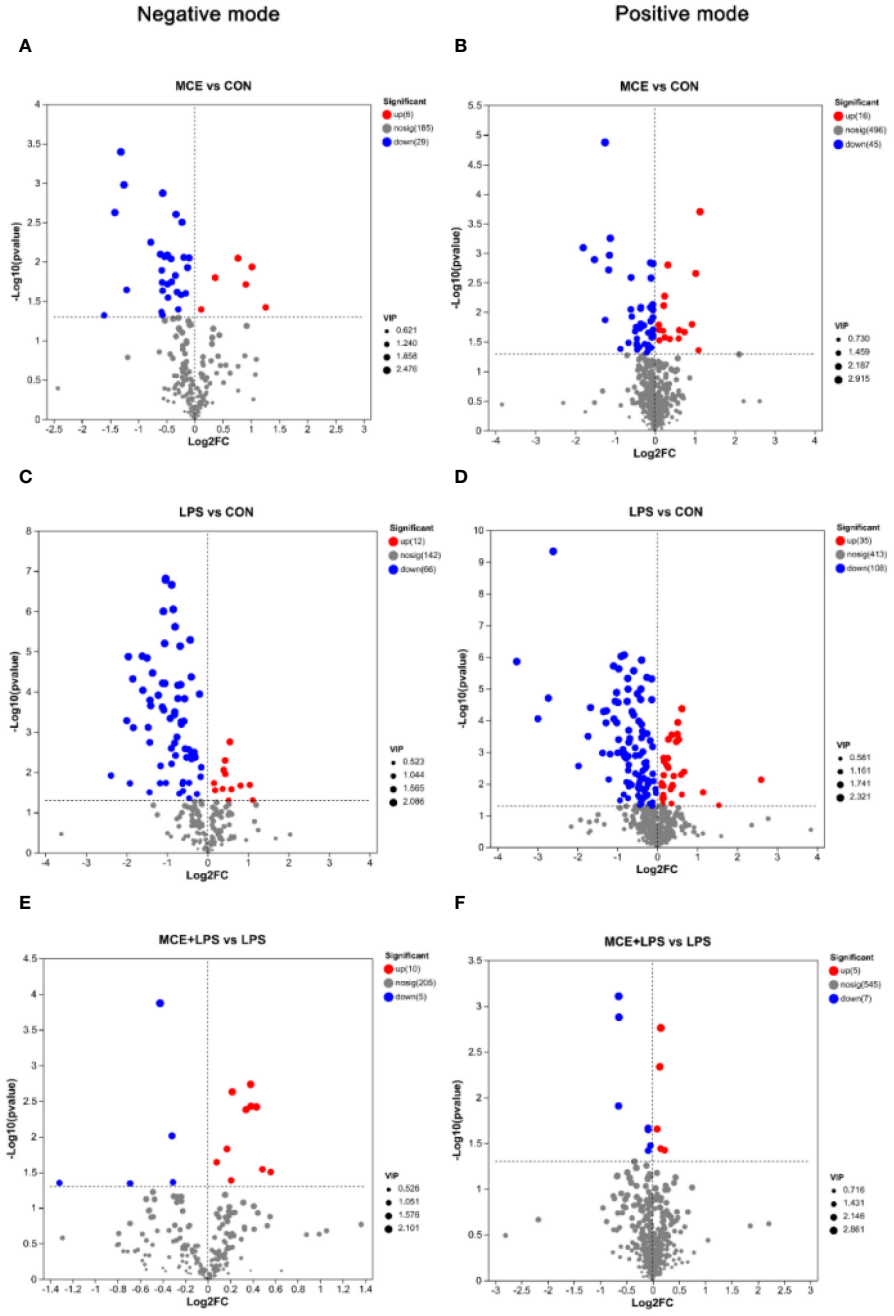
Volcano plots showing significantly up-regulated and down-regulated metabolites between different groups of broiler chickens. **(A, B)** significantly differential metabolites between the MCE and CON groups in negative and positive modes. **(C, D)** significantly differential metabolites between the LPS and CON groups in negative and positive modes. **(E, F)** significantly differential metabolites between the MCE+LPS and LPS groups in negative and positive modes. CON group, broilers received a basal diet without a LPS challenge; MCE group, broilers received a basal diet supplemented with 400 mg/kg MCE without a LPS challenge; LPS group, broilers received a basal diet and were subjected to a LPS challenge; MCE+LPS group, broilers received a basal diet supplemented with 400 mg/kg MCE and were subjected to a LPS challenge. N = 8 in each group.

The significantly differential metabolites between groups are shown in the volcano maps (**Figure 6**). As shown in **Figure 6A**, in the negative mode, compared with the CON group, the MCE group had 6 up-regulated metabolites (2,3-Dihydroxybenzoic acid, (25R)-3beta-hydroxycholest-5-en-7-one-26-oate, Ganoderic acid C1, L-Ascorbic acid, Myo-Inositol, and Pyrocatechol sulfate) and 29 down-regulated metabolites (such as Uridine, 13(S)-HODPE, Xanthosine, Xanthine, and Pseudouridine). As shown in **Figure 6B**, in the positive mode, compared with the CON group, the MCE group had 16 up-regulated metabolites, (such as Thr-Thr-Lys-Phe, Capsianoside III, Beta-Alanine, 5-Aminolevulinic acid, and Pro-Tyr-Gly) and 45 down-regulated metabolites (such as Hypoxanthine, Octadecenoylcarnitine, 13Z-Docosenamide, Tranexamic acid, and Uracil).

As shown in **Figure 6C**, in the negative mode, compared with the CON group, LPS challenge increased the levels of 12 metabolites (such as Citramalic acid, Ketosantalic acid, Ribothymidine, Pyrocatechol sulfate, and Glutaric acid) and decreased the levels of 66 metabolites, (such as Inosine, Stearic acid, Pseudouridine, L-Glutamate, and Threoninyl-Tyrosine). As shown in **Figure 6D**, in the positive mode, compared with the CON group, the LPS group had 35 up-regulated metabolites (such as (E)-2-Methylglutaconic acid, Deoxypyridinoline, 2-Naphthylamine, Stigmastanol, and 2-Deoxybrassinolide) and 108 down-regulated metabolites (such as Pantothenic Acid, Asperagenin, Octadecenoylcarnitine, Acetylcarnitine, and Val Glu Val).

As presented in **Figure 6E** and **Table 7**, in the negative mode, compared with the LPS group, the MCE+LPS group had 10 up-regulated metabolites (Gamma-Glutamylthreonine, D-altro-D-manno-Heptose, N-acetylaspartate, (1R,2S,3S,4R)-p-Menthane-2,3-diol, Stearic acid, Threoninyl-Tyrosine, L-Ribulose, L-Aspartic Acid, L-Glutamate and Myo-Inositol) and 5 down-regulated metabolites (TXB2, Lamivudine sulfoxide, Ribothymidine, 6-(3-ethenylphenoxy)-3,4,5-trihydroxyoxane-2-carboxylic acid and 5-Methoxynoracronycine). As presented in **Figure 6F** and **Table 7**, in the positive mode, compared with the LPS group, the MCE+LPS group had 5 up-regulated metabolites (Cucurbic acid, L-Serine, Corchorifatty acid A, Cyclohex-2-enone and Vinylacetylglycine) and 7 down-regulated metabolites (Sphingosine-1-phosphate, 1-Methyladenine, Oryzalexin E, Sphinganine-phosphate, Cycloalliin, (8alpha,10beta,11beta)-3-Hydroxy-4,15-dinor-1(5)-xanthen-12,8-olide and 20-Hydroxy-PGF2a).

**Table 7:** Up- and down-regulated plasma metabolites in broilers between the MCE+LPS and LPS groups in negative and positive modes.

Metabolites identified in the negative mode	Up/down	M/Z	Retention time	FC	VIP	*P*-value
Gamma-Glutamylthreonine	up	247.09	1.14	1.40	1.38	0.03
D-altro-D-manno-Heptose	up	191.06	1.02	1.27	1.71	<0.001
N-acetylaspartate	up	174.04	1.22	1.16	1.32	0.04
(1R,2S,3S,4R)-p-Menthane-2,3-diol	up	209.09	6.93	1.06	1.46	0.02
Stearic acid	up	321.22	6.91	1.13	1.51	0.01
Threoninyl-Tyrosine	up	281.11	5.13	1.47	1.40	0.03
L-Ribulose	up	149.04	1.06	1.31	1.74	<0.001
L-Aspartic Acid	up	132.03	0.99	1.35	1.72	<0.001
Myo-Inositol	up	179.06	0.90	1.16	1.78	<0.001
L-Glutamate	up	146.04	0.98	1.3	1.81	<0.001
TXB2	down	369.23	6.34	0.40	1.32	0.04
Lamivudine sulfoxide	down	266.02	3.33	0.75	2.05	<0.001
6-(3-ethenylphenoxy)-3,4,5-trihydroxyoxane-2-carboxylic acid	down	333.04	3.27	0.80	1.63	0.01
5-Methoxynoracronycine	down	372.10	2.90	0.62	1.32	0.05
Ribothymidine	down	257.08	2.82	0.81	1.31	0.04
Metabolites identified in the positive mode						
Cucurbic acid	up	245.18	6.93	1.10	2.57	<0.001
Corchorifatty acid A	up	309.20	6.78	1.11	2.74	<0.001
Vinylacetylglycine	up	144.07	2.76	1.11	2.02	0.04
Cyclohex-2-enone	up	97.07	2.14	1.06	2.16	0.02
L-Serine	up	150.01	0.91	1.17	2.00	0.04
Sphingosine-1-phosphate	down	380.26	6.72	0.64	2.78	<0.001
20-Hydroxy-PGF2a	down	371.24	6.52	0.94	2.15	0.02
Oryzalexin E	down	337.27	7.51	0.97	2.03	0.03
Sphinganine-phosphate	down	382.27	6.76	0.64	2.86	<0.001
1-Methyladenine	down	167.10	6.56	0.94	1.99	0.04
Cycloalliin	down	210.08	2.91	0.64	2.32	0.01
(8alpha,10beta,11beta)-3-Hydroxy-4,15-dinor-1(5)-xanthen-12,8-olide	down	242.18	6.09	0.94	2.17	0.02

LPS group, broilers received a basal diet and were subjected to a LPS challenge; MCE+LPS group, broilers received a basal diet supplemented with 400 mg/kg MCE and were subjected to a LPS challenge; M/Z, mass to charge ratio; FC, fold change; VIP, variable important in projection; TXB2, thromboxane B2; 20-Hydroxy-PGF2a: 20-hydroxy prostaglandin F2a. N = 8 in each group.

### KEGG enrichment of differential metabolites

**Figure 7:**
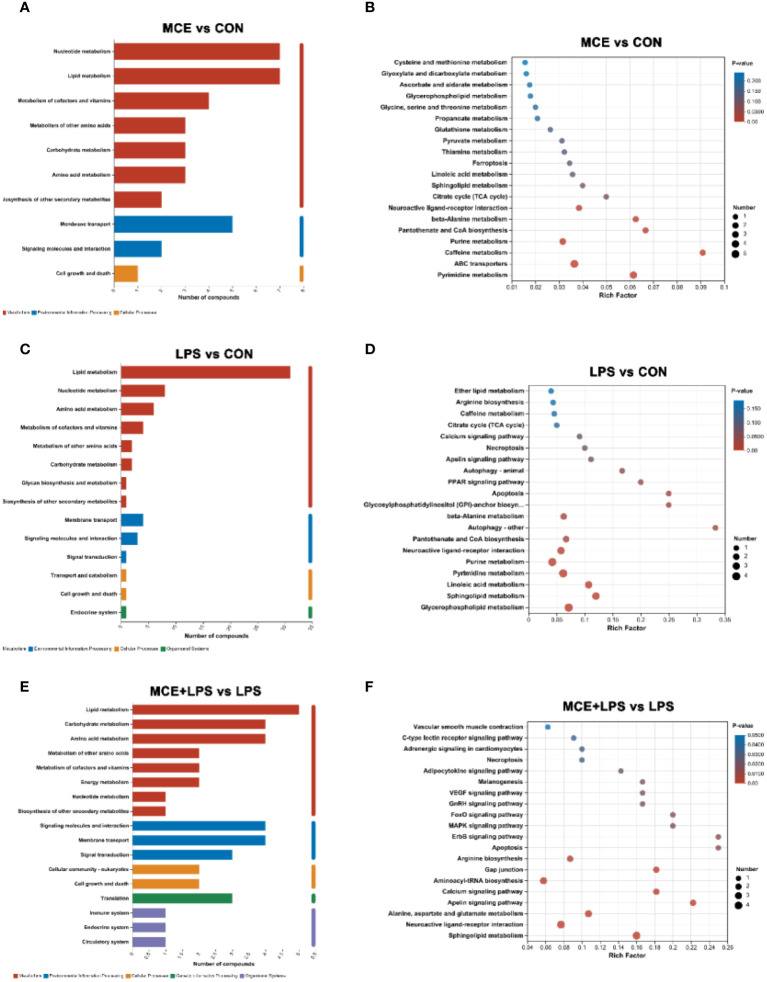
Effects of Macleaya cordata extract (MCE) on KEGG pathways enrichment for differential plasma metabolites in broiler chickens. The first and second **(A)** and third **(B)** classification categories of the KEGG pathway between MCE and CON groups. The first and second **(C)** and third **(D)** classification categories of the KEGG pathway between LPS and CON groups.The first and second **(E)** and third **(F)** classification categories of the KEGG pathway between MCE+LPS and LPS groups. CON group, broilers received a basal diet without a LPS challenge; MCE group, broilers received a basal diet supplemented with 400 mg/kg MCE without a LPS challenge; LPS group, broilers received a basal diet and were subjected to a LPS challenge; MCE+LPS group, broilers received a basal diet supplemented with 400 mg/kg MCE and were subjected to a LPS challenge. N = 8 in each group.

As presented in **Figure 7**, the KEGG metabolic pathway enrichment analysis demonstrated that significantly differential metabolites between the MCE and CON groups were enriched in Nucleotide metabolism, Lipid metabolism, Metabolism of cofactors and vitamins, Metabolism of other amino acids, Carbohydrate metabolism, Amino acid metabolism and others. MCE supplementation altered the pathways of Pyrimidine metabolism, ABC transporters, Caffeine metabolism, Purine metabolism, Pantothenate and CoA biosynthesis, beta-Alanine metabolism and others.

The significantly differential metabolites between the LPS and CON groups were enriched in Lipid metabolism, Nucleotide metabolism, Amino acid metabolism, Metabolism of cofactors and vitamins, Metabolism of other amino acids, Carbohydrate metabolism and others. Compared with the CON group, LPS challenge altered the pathways of Glycerophospholipid metabolism, Sphingolipid metabolism, Linoleic acid metabolism, Pyrimidine metabolism, Purine metabolism, Neuroactive ligand-receptor interaction, Pantothenate and CoA biosynthesis and others.

The significantly differential metabolites between the MCE+LPS and LPS groups were enriched in Lipid metabolism, Carbohydrate metabolism, Amino acid metabolism, Metabolism of other amino acids, Metabolism of cofactors and vitamins, Energy metabolism, and others. Compared with the LPS group, MCE supplementation altered the pathways of Sphingolipid metabolism, Neuroactive ligand-receptor interaction, Alanine, aspartate and glutamate metabolism, Apelin signaling pathway, Calcium signaling pathway, Aminoacyl-tRNA biosynthesis, Gap junction, Arginine biosynthesis, Apoptosis, MAPK signaling pathway and others.

### Correlation analysis between gut microbiota and metabolites

**Figure 8:**
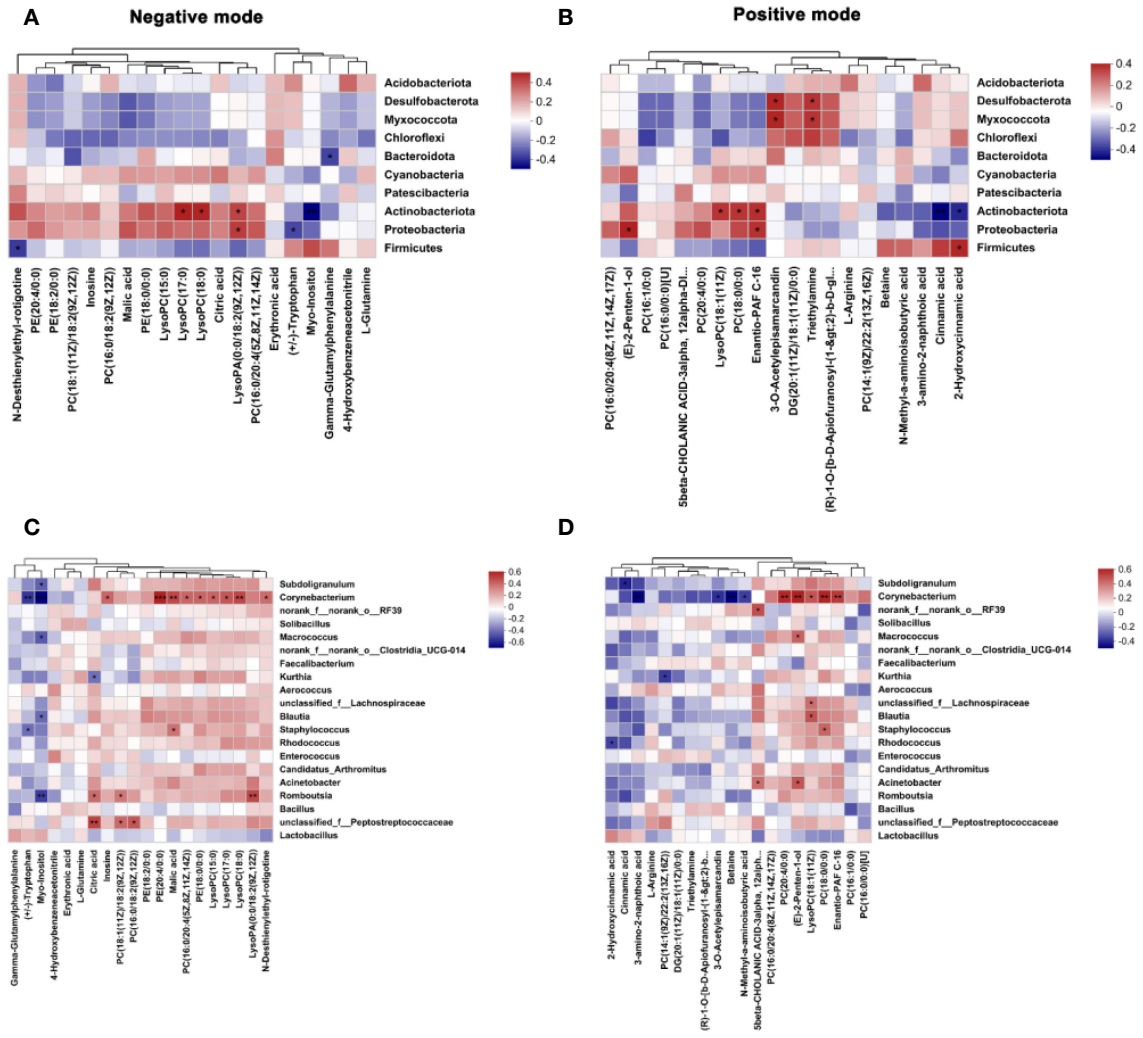
Spearman correlation analysis of gut microbiota and plasma metabolites in broiler chickens. **(A, B)** correlation between the top 10 phyla of ileal microbiota and the top 20 differential plasma metabolites in the negative and positive modes. **(C, D)** correlation between the top 20 genera of ileal microbiota and top 20 differential plasma metabolites in the negative and positive modes. CON group, broilers received a basal diet without a LPS challenge; MCE group, broilers received a basal diet supplemented with 400 mg/kg MCE without a LPS challenge; LPS group, broilers received a basal diet and were subjected to a LPS challenge; MCE+LPS group, broilers received a basal diet supplemented with 400 mg/kg MCE and were subjected to a LPS challenge. Significance was set at *P* < 0.05. *P < 0.05, **P < 0.01, ***P < 0.001. N = 8 in each group.

As shown in **Figure 8A**, at the phylum level, in the negative mode, the relative abundance of *Firmicutes* was negatively correlated with N-Desthienylethyl-rotigotine. A negative correlation was observed between the relative abundance of *Proteobacteria* and (+/-)-Tryptophan. The relative abundance of *Actinobacteriota* was positively correlated with lysophosphatidylcholine (LysoPC)(17:0) and LysoPC(18:0) and was negatively correlated with Myo-Inositol. The relative abundance of *Bacteroidota* showed a negative correlation with gamma-glutamylphenylalanine. As shown in **Figure 8B**, in the positive mode, A positive correlation was observed between the relative abundance of *Firmicutes* and 2-Hydroxycinnamic acid. The relative abundance of *Actinobacteriota* was positively correlated with LysoPC(18:1(11Z)) and phosphatidylcholine (PC)(18:0/0:0) and was negatively correlated with Cinnamic acid and 2-Hydroxycinnamic acid. The relative abundance of *Desulfobacterota* exhibited a positive correlation with 3-O-Acetylepisamarcandin and Triethylamine.

As presented in **Figure 8C**, at the genus level, in the negative mode, the relative abundance of *Peptostreptococcaceae* was positively correlated with PC(18:1(11Z)/18:2(9Z,12Z)) and PC(16:0/18:2(9Z,12Z)). The relative abundance of *Romboutsia* showed a positive correlation with Citric acid. The relative abundance of *Corynebacterium* was positively correlated with inosine, phosphatidylethanolamine (PE)(20:4/0:0), PC(16:0/20:4(5Z,8Z,11Z,14Z)), PE(18:0/0:0), and LysoPC(15:0) and was negatively correlated with (+/-)-Tryptophan and Myo-Inositol. As shown in **Figure 8D**, in the positive mode, the relative abundance of *Rhodococcus* was negatively correlated with 2-Hydroxycinnamic acid. A positive correlation was observed between the relative abundance of *Staphylococcus* and PC (18:0/0:0). The relative abundance of *Kurthia* diaplayed a negative correlation with PC(14:1(9Z)/22:2(13Z,16Z)). The relative abundance of *Corynebacterium* was positively correlated with PC(20:4/0:0), LysoPC(18:1(11Z)) and PC(18:0/0:0), and was negatively correlated with Betaine.

## Discussion

In this study, MCE supplementation significantly increased the ADFI during days 0-14, and, the ADG during days 15-21 was higher in the MCE+LPS group than in the LPS group. The results are in agreement with previous studies. Dietary supplementation of 350 mg/kg MCE effectively alleviated the necrotic enteritis-induced reduction of growth performance in broiler chickens (19). Diet with 0.6 mg/kg of MCE containing protopine and allotypotopine increased the final body weight and ADG and decreased F/G of broiler chickens from d 0 to 42 (28). It was reported that dietary supplementation of 50 mg/kg MCE could enhance the growth performance and intestinal morphology of early-weaned piglets (13). Sanguinarine and chelerythrine have similar structures to aromatic amino acids, which can irreversibly inhibit the activity of intestinal aromatic amino acid decarboxylase (29). Sanguinarine has been proved to promote protein retention by lowering intestinal decarboxylation of aromatic amino acids and increases feed intake by regulating the Trp-serotonin pathway, thus improving animal growth (17, 30). These responses may explain the improved growth performance of broiler chickens by MCE supplementation in this study.

T-AOC represents the capacity of the enzymatic and non-enzymatic antioxidant system in the body (31). CAT, GSH-Px and SOD are important antioxidant enzymes involved in eliminating superoxide anions and hydrogen peroxide during oxidative damage (31, 32). The level of MDA reflects the degree of lipid peroxidation in the body and is an indicator of initial cellular membrane damage (33). Our study revealed that dietary MCE supplementation increased the T-AOC and CAT activity while decreasing MDA levels in the jejunum. Similarly, dietary supplementation of 200 mg/kg MCE observably alleviated oxidative stress induced by enterotoxigenic *Escherichia coli* in mice, as illustrated by lower levels of MDA and increased activities of CAT and GSH-Px (18). It has been reported that sanguinarine not only decreased the ROS level induced by LPS, but also increased the activity of antioxidant enzymes by activating the Nrf2 signaling pathway in mouse mammary epithelial cells (34). Moreover, sanguinarine can inhibit the activities of nicotinamide adenine dinucleotide phosphate oxidase 2, thereby inhibiting the conversion of NADPH to ROS and improving the antioxidant capacity (35).

The intestinal morphology is a crucial indicator for gut health and intestinal absorption capacity. This study demonstrated that diets supplemented with MCE could effectively increase the ileal villus height and VCR in broiler chickens. Similarly, Song et al. (19) has discovered that the diet supplemented with 350 mg/kg MCE significantly improved the growth performance, increased the jejunal villus height and VCR and decreased the intestinal lesion score in broiler chickens with necrotic enteritis. Dietary sanguinarine supplementation at 0.7 mg/kg improved growth performance and increased the villus height and VCR of the duodenum, jejunum and ileum in yellow feathered broiler chickens at 28 days of age (17). Piglets fed a basal diet supplemented with 50 mg/kg MCE exhibited a lower crypt depth of the jejunum, higher villus height of the ileum, and higher VCR of the jejunum and ileum than the animals fed the basal diet (13). According to the findings of this study, MCE may enhance growth performance by improving intestinal morphology, thus facilitating efficient nutrient absorption.

IL-1β is the main pro-inflammatory mediator of systemic inflammatory responses (36). IL-6 is synthesized in the early period of inflammation (37). IL-8 can cause mucosal damage via the release of ROS, metalloproteinases and cytokines (38). IL-17 plays a pro-inflammatory role by promoting the secretion of chemokines (39). This study showed that dietary MCE supplementation decreased the mRNA expression of pro-inflammatory factors *IL-1β*, *IL-8* and *IL-17* in LPS-challenged broiler chickens, which indicated that MCE effectively suppressed the intestinal inflammatory responses induced by LPS challenge. Similar study has reported that pretreatment with MCE dramatically decreased the elevated mRNA expression of pro-inflammatory cytokines, such as *IL-1α, IL-1β* and *IL-8* in porcine alveolar macrophages induced by *Glaesserella parasuis* (15). Previous research also found that sanguinarine and chelerythrine are responsible for the anti-microbial, anti-inflammatory and immunomodulatory properties of Macleaya cordata (40). Sanguinarine and chelerythrine displayed anti-inflammatory activity by promoting nuclear translocation of the glucocorticoid receptor and inhibiting activation of NF-κB (14, 15).

*Staphylococcus* can secrete various toxins and pro-inflammatory factors, alter the junctions between epithelial cells, disrupt the intestinal barrier, and increase intestinal permeability (41, 42). Moreover, *Staphylococcus* has strong drug resistance and can survive inside a variety of immune cells and enter the blood, causing infections of the gut, lung, and other tissues (43–45). *Actinobacteriota* are Gram-positive pathogens with high base pair of G+C, such as *Corynebacterium* and *Nocardia* (46). *Streptococcus* can produce streptolysin O, a pore-forming toxin that can influence the intestinal health (47). The LefSe results demonstrated that *Staphylococcus*, *Actinobacteriota* and *Streptococcus* were the taxonomic biomarkers in broiler chickens challenged with LPS, suggesting that the stimulation of LPS can lead to an increase in the relative abundance of potentially harmful bacteria in the intestine of broiler chickens. *Blautia* can use glucose to produce acetic acid, succinic acid, and lactic acid, and increase the content of volatile fatty acids (48). Besides, it can use carbon dioxide, carbon monoxide, hydrogen, and cellulose that cannot be used by the host as an energy source to regulate intestinal pH and maintain intestinal homeostasis (49). The LefSe results showed that birds in the MCE group has higher relative abundance of *Blautia* compared with other other groups, which may be related to the alleviation of intestinal inflammation and improvement of intestinal morphology.

*Firmicutes* are the dominant phylum of bacteria in broiler chickens. It has been reported that mice colonized with microbiota abundant in *Firmicutes* from healthy human fecal samples exhibited downregulation of the TH17 pathway and colonic inflammation (50). In this study, MCE supplementation increased the relative abundance of *Firmicutes*, which was similar to the studies of Song et al. (19) in a necrotic enteritis model and Wang et al. (51) in a chronic heat stress model. *Lactobacillus* can inhibit the activity of pathogenic bacteria (such as *Escherichia* coli and *Staphylococcus aureus*) and regulate the activity of immune cells and epithelial cells, thereby improving host immunity and intestinal barrier function (52). The present study showed that MCE supplementation increased the relative abundance of *Lactobacillus*. This was in agreement with the findings observed in mice, where MCE administration alleviated the decreased *Lactobacillus* population induced by heat stress (53). Meanwhile, dietary MCE supplementation can increase the abundance of *Lactobacillus* and adjust the intestinal pH, which is beneficial to the intestinal luminal environment in early-weaned piglets (13). *Actinobacteriota*, *Peptostretococcaceae* and *Rhodococcus* can promote inflammation by releasing pathogenic toxins such as diphtheria toxin (54). It has been reported that *Actinobacteriota* is highly associated with ulcerative colitis, Crohn’s disease, immunodeficiency, and other diseases (55). Our study showed that MCE could alleviate the increase in the relative abundance of *Actinobacteriota*, *Peptostretococcaceae* and *Rhodococcus* caused by LPS, thereby suppressing intestinal inflammation.

The results of differential metabolite analysis and KEGG enrichment analysis between the MCE+LPS and LPS groups showed that the different metabolites were mainly enriched in Lipid metabolism, Nucleotide metabolism, Amino acid metabolism, and Membrane transport. In Amino acid metabolism pathway, the MCE+LPS group significantly increased the levels of L-aspartic acid, L-glutamate, L-serine, β-alanine, and 5-Hydroxy-L-tryptophan compared with the LPS group. L-aspartic acid can inhibit the formation of cell membranes in *Staphylococcus* aureus, and regulate leukocyte phagocytosis and the immune response (56, 57). In addition, it can promote the entry of argininosuccinic acid into the tricarboxylic acid cycle and enhance cell survival (58). L-glutamate is the precursor of glutathione and plays an antioxidant role. It is also the precursor of γ-aminobutyric acid (GABA), which acts as a signaling molecule in brain cells, islet cells and intestinal cells. Moreover, glutamate is the only amino acid that can stimulate afferent gastric vagal nerves (59). L-serine can bind to pyruvate kinase to produce pyruvate and affect glucose metabolism, and it is a necessary amino acid for ceramide synthesis (60). In addition, 5-Hydroxy-L-tryptophan can enhance glucose and lipid metabolism and stimulate the proliferation of T cells and B lymphocytes through the synthesis of 5-hydroxytryptamine (61–64). β-alanine is the rate-limiting precursor for the synthesis of carnosine synthesis, and it can exert antioxidant and neuroprotective effects by regulating carnosine synthesis (65–67). These results suggest that MCE supplementation can regulate the levels of related metabolites and alter amino acid metabolism.

In the present study, hypoxanthine, uracil and deoxyguanosine are enriched in the nucleotide metabolism pathway. Hypoxanthine produces ROS during metabolism, which may cause oxidative stress, trigger caspase-3-induced apoptosis, and increase inflammation (68). Uracil is formed by mismatch due to spontaneous deamination of cytosine (69). Deoxyguanosine and pseudouridine are the biomarkers for oxidative DNA damage and prostate cancer, respectively (70). In this study, we found that dietary MCE supplementation could decrease the levels of these metabolites and regulate nucleotide metabolism. In addition, our study showed that 13-(S)-HODPE, PC and PE were key metabolites in lipid metabolism. It is reported that 13-(S)-HODPE can increase the production of ROS and promote the expression of apoptotic genes such as caspase-3 and p21, thus promoting cell apoptosis (71). The present study showed that MCE decreased the level of 13-(S)-HODPE, suggesting that MCE may inhibit oxidant stress by reducing the 13-(S)-HODPE level. The present study demonstrated that MCE supplementation significantly reduced the level of 13-(S)-HODPE. PC and PE are the most abundant phospholipids in all mammalian cell membranes, and the ratio of PC to PE is a key factor for low-density lipoprotein metabolism and energy metabolism in cellular organelles, and it plays an important role in gut and liver health (72). This study showed that MCE supplementation decreased the levels of both PC and PE, suggesting that MCE supplementation could regulate the lipid metabolic pathway and participate in the regulation of gut inflammation.

The results of Spearman correlation analysis of gut microbiota and plasma metabolites showed that the relative abundance of *Firmicutes* was positively correlated with the level of 2-hydroxycinnamic acid, a well-known antioxidant (73, 74), suggesting that *Firmicutes* could exert antioxidant function by increasing the level of 2-Hydroxycinnamic acid. *Corynebacterium* plays a pathogenic role by secreting phospholipase D (PLD) and diphtheria toxin, and PLD can activate NF-κB in epithelial cells, release inflammatory factors such as IL-6, and increase vascular permeability (75). In addition, *Corynebacterium* is closely associated with inflammatory diseases, such as bacteriemia and endocarditis (76). Our results showed that the relative abundance of *Corynebacterium* was negatively correlated with (+/-)-tryptophan and myo-inositol and positively correlated with inosine, suggesting that *Corynebacterium* could alter the levels of related metabolites, thereby regulating amino acid and nucleotide metabolic pathways.

## Conclusion

In conclusion, dietary MCE supplementation at 400 mg/kg benefited the growth performance and alleviated the intestinal injury in broiler chickens challenged with LPS, which might be closely related to the modulation of gut microbiota and plasma metabolites. MCE supplementation could increase the relative abundance of potentially probiotic bacteria (such as *Blautia* and *Lactobacillus*) and decrease the relative abundance of potentially pathogenic bacteria (such as *Actinobacteriota*, *Peptostretococcaceae*, and *Rhodococcus*) in the gut. Moreover, MCE supplementation could alter the composition of metabolites and regulate many metabolic pathways, mainly including Lipid metabolism, Amino acid metabolism, and Nucleotide metabolism in broiler chickens. This study provides a valuable reference for nutritional regulation to prevent the gut damage induced by immune stress in broiler chickens.

## Data availability statement

The original contributions presented in the study are publicly available. This data can be found here: Sequence Read Archive of NCBI, accession number PRJNA1087016.

## Ethics statement

The animal study was approved by Animal Care and Use Committee of Qingdao Agricultural University (No. DKY20220513). The study was conducted in accordance with the local legislation and institutional requirements.

## Author contributions

XW: Data curation, Investigation, Methodology, Writing – original draft. TZ: Data curation, Investigation, Methodology, Writing – review & editing. WL: Project administration, Supervision, Writing – review & editing. MZ: Project administration, Supervision, Writing – review & editing. LZ: Methodology, Software, Writing – review & editing. NW: Methodology, Software, Writing – review & editing. XZ: Methodology, Software, Writing – review & editing. BZ: Funding acquisition, Investigation, Project administration, Supervision, Writing – review & editing.
